# CREG ameliorates the phenotypic switching of cardiac fibroblasts after myocardial infarction via modulation of CDC42

**DOI:** 10.1038/s41419-021-03623-w

**Published:** 2021-04-06

**Authors:** Dan Liu, Xiaoxiang Tian, Yanxia Liu, Haixu Song, Xiaoli Cheng, Xiaolin Zhang, Chenghui Yan, Yaling Han

**Affiliations:** Cardiovascular Research Institute and Department of Cardiology, General Hospital of Northern Theater Command, Shenyang, China

**Keywords:** Mechanisms of disease, Heart failure

## Abstract

Phenotype switching of cardiac fibroblasts into myofibroblasts plays important role in cardiac fibrosis following myocardial infarction (MI). Cellular repressor of E1A-stimulated genes (CREG) protects against vascular and cardiac remodeling induced by angiotensin-II. However, the effects and mechanisms of CREG on phenotype switching of cardiac fibroblasts after MI are unknown. This study aimed to investigate the role of CREG on the phenotype switching of cardiac fibroblasts following MI and its mechanism. Our findings demonstrated that, compared with littermate control mice, cardiac function was deteriorated in CREG^+/−^ mice on day 14 post-MI. Fibrosis size, αSMA, and collagen-1 expressions were increased in the border regions of CREG^+/−^ mice on day 14 post-MI. Conversely, exogenous CREG protein significantly improved cardiac function, inhibited fibrosis, and reduced the expressions of αSMA and collagen-1 in the border regions of C57BL/6J mice on day 14. In vitro, CREG recombinant protein inhibited αSMA and collagen-1 expression and blocked the hypoxia-induced proliferation and migration of cardiac fibroblasts, which was mediated through the inhibition of cell division control protein 42 (CDC42) expression. Our findings could help in establishing new strategies based on the clarification of the role of the key molecule CREG in phenotype switching of cardiac fibroblasts following MI.

## Introduction

Myocardial infraction (MI) is a major public health problem^1^, and its mortality rate is decreasing with improved treatment strategies. However, MI-related heart failure remains a major problem without established targeted therapies. Post-MI cardiac remodeling results from several changes in the shape and function of the left ventricle, which ultimately lead to heart failure secondary to pathological changes of cardiac fibrosis^1–3^.

Cardiac fibroblasts are major homeostatic modulators of excessive extracellular matrix (ECM). Due to pathological stimuli, such as hypoxia, cardiac fibroblasts are activated and transformed into cardiac myofibroblasts^4^, which play important roles in cardiac fibrosis^5,6^. These fibroblasts differentiate into myofibroblasts, proliferate, and secrete excess ECM, such as collagen and matrix metalloproteinase, which result in abnormal myocardial stiffness, cardiac fibrosis, and heart failure^7,8^. Although previous studies suggest inflammation and autophagy play a vital role in cardiac fibroblast activation^9,10^, no effective therapies targeting phenotypic transformation are available. Therefore, identifying the key preventive method of the phenotypic transformation of cardiac fibroblasts after MI could provide new strategies for the prevention and treatment of heart failure following MI.

Cellular repressor of E1A-stimulated genes (CREG) is a secreted glycoprotein that is widely expressed in adult tissues and cells^11–14^. CREG is an important mediator of vascular remodeling in response to angiotensin-II (Ang-II)^13^. Studies have indicated that CREG is an important myocardial protective factor^15–17^. CREG could promote cardiomyogenic differentiation by interacting with Sec8^14^. CREG ameliorates cardiac fibrosis induced by Ang-II by regulating Rab7^18^. However, whether CREG could ameliorate post-MI cardiac fibrosis by regulating cardiac myofibroblast activation remains unknown. Therefore, in this study, we aimed to investigate the role of CREG in cardiac fibrosis and post-MI phenotypic transformation of cardiac fibroblasts and clarify the underlying molecular mechanisms.

## Materials and methods

### Animals

CREG heterozygous mice (CREG^+/−^) and age-matched littermates, male C57BL/6J mice (8 weeks) were generated or purchased from GemPharmatech Co., Ltd (China). Besides, cardiomyocyte-specific CREG knockout mice (CREG-CKO) were also generated and verified (GemPharmatech Co., Ltd). Briefly, CREG-flox/flox mice were crossed with transgenic mice expressing Cre recombinase under cardiac muscle-specific α-MHC promoter. Genomic DNA isolated tails from CREG-flox/flox and CREG-CKO mice were used to identify mouse genotypes. All male mice were used in a C57BL/6J background and were housed in temperature-controlled cages with 12-h light-dark cycle and provided free access to food and water.

### MI protocol

The MI model has been described previously^19^. CREG^+/−^ mice and littermate controls (male, 8 weeks) were randomly divided into sham group (*n* = 10) and MI group (*n* = 22 for littermate controls and *n* = 17 for CREG^+/−^ mice), CREG-CKO mice and flox/flox mice (male, 8 weeks) were randomly divided into sham group (*n* = 10) and MI group (*n* = 10). C57BL/6J mice (male, 8 weeks) were randomly divided into sham (*n* = 10), MI (*n* = 22), MI + vehicle (*n* = 22), and MI + CREG groups (*n* = 25). In MI + CREG group, CREG recombinant protein (rCREG, 150 μg/kg/d, Abcam, USA)^13,17^ was administered continuously via subcutaneously implanted osmotic pump (Alzet, USA) within 5 min of MI and sustained the whole experiment for 14 days. The MI + vehicle group was administered equivalent PBS solution via an osmotic pump. The endpoint was 14 days post-MI. All animal procedures conformed to the “Guide for the Care and Use of Laboratory Animals” published by NIH and the Animal Care and Use Committee of the General Hospital of Northern Theater Command.

### Determination of cardiac function

Cardiac function was evaluated using small animal ultrasound (Vevo 2100, Canada). Cardiac dimensions were studied by M-mode echocardiography. Left ventricular end-diastolic diameter and end-systolic diameter were measured on the left ventricular long-axis view. Ejection fraction (EF%) and fractional shortening (FS%) were calculated by computer algorithms. All of these measurements were performed in a blinded manner.

### Masson’s trichrome staining and Immunohistochemistry

The heart sections were dehydrated, embedded in paraffin, and 3-μm-thick sections cut using microtome were mounted on slides. Five sections were used for Masson’s trichrome staining to measure the infarct size in the infarct region and fibrosis in the border zone of the myocardium. The stained sections were scanned using a microscope (Zeiss, Germany), and the infarct and fibrosis areas were estimated using Image-Pro Plus v6.0 software.

In addition, the sections were stained for α-smooth muscle actin (αSMA, Abcam) and collagen-1 (Abcam) in the border zone using an immunohistochemistry kit. The sections were photographed using a microscope (Zeiss).

### Flow cytometry

Two-color flow cytometry (Lin^−^:Ter119^−^CD45^−^CD31^−^, GP38^+^) was used to identify cardiac fibroblasts from the adult mouse heart as previously described^20^. Briefly, the hearts in the sham and MI groups were perfused with langendorff fluid for 5 min and digested with a mixture of trypsin and collagenase for 15 min. The hearts were cut into small pieces and the digestion was terminated using the serum. These tissues were filtered through a 10-μm cell strainer followed by centrifugation, and the supernatants were washed with phosphate buffer saline (PBS). Single-cell suspension was obtained by resuspending the cell pellet in PBS buffer. Cells were incubated for 30 min with the appropriate fluorochrome-labeled antibodies (Ter119-PE, CD45-PE, CD31-PE, and GP38-APC [eBioscience, USA]). The cells were analyzed using a flow cytometer. Three independent experiments were performed.

### Cell culture

Cardiac fibroblasts were separated from the adult heart and 1–3 day-old neonatal heart^21^, and treated with hypoxia (1% O_2_) for 24 h. To establish CREG knockdown cell, CREG siRNA (si-CREG, Santa Cruz Biotechnology, USA) was transfected into cells using lipofectamine RNAi max (Thermo fisher Scientific, USA). CREG recombinant protein (rCREG, 2.5 μg/ml or 5 μg/ml, Abcam) was added to the cultured medium for 24 h. CREG siRNA or rCREG was delivered into cells to examine the effect of CREG on cardiac fibroblast activation under hypoxia. To clarify the role of CDC42 on the functions of cardiac fibroblasts, CDC42 overexpressed adenovirus (adCDC42, OBIO Technology, China) was added to the cell for 24 h and followed with hypoxia for an additional 24 h. In rescue experiments, cells were given rCREG and adCDC42 followed by hypoxia for 24 h. The cells were collected at 24 h after hypoxia. Three independent experiments were performed.

### Quantitative real-time polymerase chain reaction (Real-time PCR)

RNA from cardiac fibroblasts and heart tissue was extracted using an RNA extraction kit (Promega, USA). The mRNA expressions of CREG and CDC42 in cardiac fibroblasts were examined by real-time PCR. Primer for CREG was: CTTCGCGGACATCATCTCAAT (Forward), GTCAGCGTAGCCTCTG GATTT (Reverse); Primer for CDC42 was: GCCTGAGATAACTCACCACTGTCC (Forward), GGCAGAGCACTCCACATACTTGAC (Reverse); Primer for GAPDH was: AGGTCGGTGTGAACGGATTTG (Forward), GGGGTCGTTGATGGCAACA (Reverse). Amplification and detection were performed using a CFX96 Real-Time System (Bio-Rad, USA).

### Western blotting

The proteins of heart tissue or cardiac fibroblasts were prepared in RIPA lysis (Thermo fisher Scientific, USA). The following antibodies including CREG (Abcam), αSMA (Abcam), collagen-1 (Abcam), cleaved caspase 3 (Cell Signaling Technology, USA), Bax (Cell Signaling Technology, USA), proliferating cell nuclear antigen (PCNA, Abcam), CDC42 (Abcam), Rac1 (Abcam) and RhoA (Abcam), p21-activated kinase 1 (PAK1, Cell Signaling Technology), phosphorylated-PAK1 (Abcam) and insulin-like growth factor-2 receptor (IGF2R, Abcam) were used. β-actin was as the internal reference (Abcam).

### Immunoprecipitation (IP)

The protein of mouse 3T3 fibroblasts (1000 μg) was incubated with CREG, CDC42, or IgG antibody (Abcam) and protein A/G beads (Thermo fisher Scientific) overnight at 4 °C. The beads were washed 3 times and re-suspended in a 2× loading buffer. The expression of CREG and CDC42 were examined in the IP supernatant using western blotting. Experiments were performed in triplicate.

### Mass spectrometry

The heart tissue of CREG^+/−^ mice and age-matched littermate control mice (8 weeks) was delivered for mass spectrum (Beijing Genomics institution, China), which was performed to screen for proteins with differential expression in the myocardium of CREG^+/−^ mice and littermate control mice.

### Pull-down assay

CDC42 activity was determined using CDC42 activation assay biochem kit (Cytoskeleton)^22^. Briefly, GTPγS (positive control) or GDP (negative control) was performed. Affinity precipitation of activated CDC42 was performed using PAK-PBD beads, and CDC42 signal in the samples was subsequently analyzed using western blotting.

### Immunofluorescence staining

Heart tissues or cardiac fibroblasts were fixed with 4% paraformaldehyde, incubated with the primary antibodies against CREG (Sigma Aldrich, USA), CDC42 (Abcam), αSMA (Abcam), collagen-1 (Abcam), and PCNA (Abcam) for overnight, and incubated with secondary antibodies (Thermo fisher Scientific). Cell nuclei were stained with DAPI. Samples were scanned using confocal microscopy (Zeiss).

### Cell counting Kit-8 (CCK8) assay

Cells were plated in 96-well format. Cells were given rCREG (5 μg/ml, Abcam), adCDC42 (OBIO Technology), or si-CREG, and followed by hypoxia for 24 h. CCK8 (Beyotime, China) was incubated for 2 h. The optical density value was measured at 450 nm. Three independent experiments were performed.

### Wound healing assay

Cells were plated in a 6-well format. Cells were given rCREG (5 μg/ml, Abcam) or adCDC42 (OBIO Technology) for 24 h. A straight scratch was marked using a pipette tip. Cells were stimulated with hypoxia for an additional 12 h after a straight scratch. The cells at 0 h and 12 h after scratching were photographed using a ×10 microscope (Zeiss).

### Statistical analysis

Data analysis was performed using SPSS v22.0 (IBM Inc., Armonk, NY, USA). Data were expressed as mean ± standard error of the mean (SEM). Differences between the two groups were compared using unpaired Student’s *t*-tests. Differences between three or more groups were compared using one-way analysis of variance (ANOVA). Statistical significance was defined as *P* < 0.05.

## Results

### Decreased CREG expression was associated with cardiac myofibroblast activation in the myocardium of C57BL/6J mice following MI

**Fig. 1:**
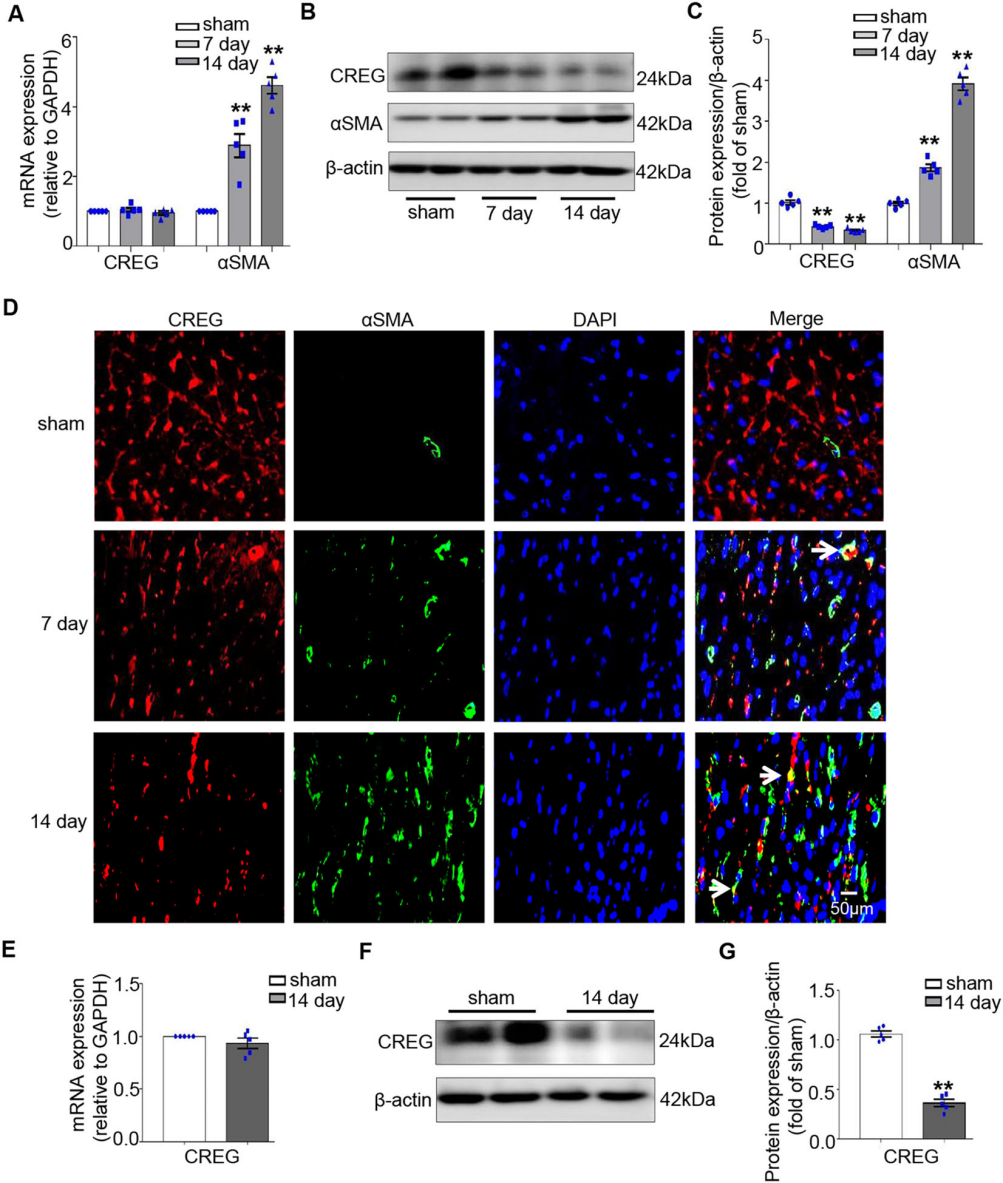
CREG expression was decreased in the border zone of C57BL/6J mice after MI. **A** Real-time PCR of *CREG* and *αSMA* mRNA expression in the border zone of C57BL/6J mice on post-MI days 7 and 14 (*N* = 5 per group). **B**, **C** Western blotting of CREG and αSMA protein expression in the border zone of C57BL/6J mice on post-MI days 7 and 14 (*N* = 5 per group). **D** Immunofluorescence staining of CREG (red) and αSMA (green) expression in the border zone of myocardium in C57BL/6J mice on post-MI days 7 and 14 (*N* = 3 per group). The arrow represented the co-location of CREG with αSMA. **E** Real-time PCR for *CREG* mRNA in primary cardiac fibroblast of MI group and sham group (*N* = 5 per group). **F**, **G** Western blotting for CREG in primary cardiac fibroblast of MI group and sham group (*N* = 5 per group). **P < 0.01 vs. sham group.

To investigate their association with post-MI cardiac myofibroblast activation, the expressions of CREG and αSMA, a marker of cardiac myofibroblast activation, were evaluated in the border zone myocardium of C57BL/6L mice on days 7 and 14. *CREG* mRNA level was unchanged following MI (Fig. 1A); however, *αSMA* mRNA was increased (*P* < 0.01, Fig. 1A). CREG protein expression was significantly decreased in the border zone, which was parallel to the increase in αSMA protein expression (*P* < 0.01, Fig. 1B, C). Furthermore, in the sham group, CREG was highly expressed in the myocardium, and αSMA was only expressed on the blood vessels. However, in the border zone, CREG expression was significantly decreased and αSMA-marked cardiac myofibroblasts were increased (Fig. 1D). Therefore, a negative correlation was observed between decreased CREG expression and cardiac myofibroblasts activation post-MI.

CREG is ubiquitously expressed in differentiated tissue and cells; therefore, these above results did not reflect that whether CREG was decreased in fibroblasts. To test specific reduction of CREG in fibroblasts, CREG expression in primary cardiac fibroblasts of C57BL/6J mice on post-MI day 14 was evaluated using real-time PCR and western blotting. The results revealed that CREG protein expression was significantly decreased in cardiac fibroblasts following MI (*P* < 0.01, Fig. 1E–G). These results indicated that CREG expression in cardiac fibroblasts was downregulated following MI, and was negatively correlated to cardiac myofibroblasts activation.

### CREG deficiency impaired cardiac function after MI

**Fig. 2:**
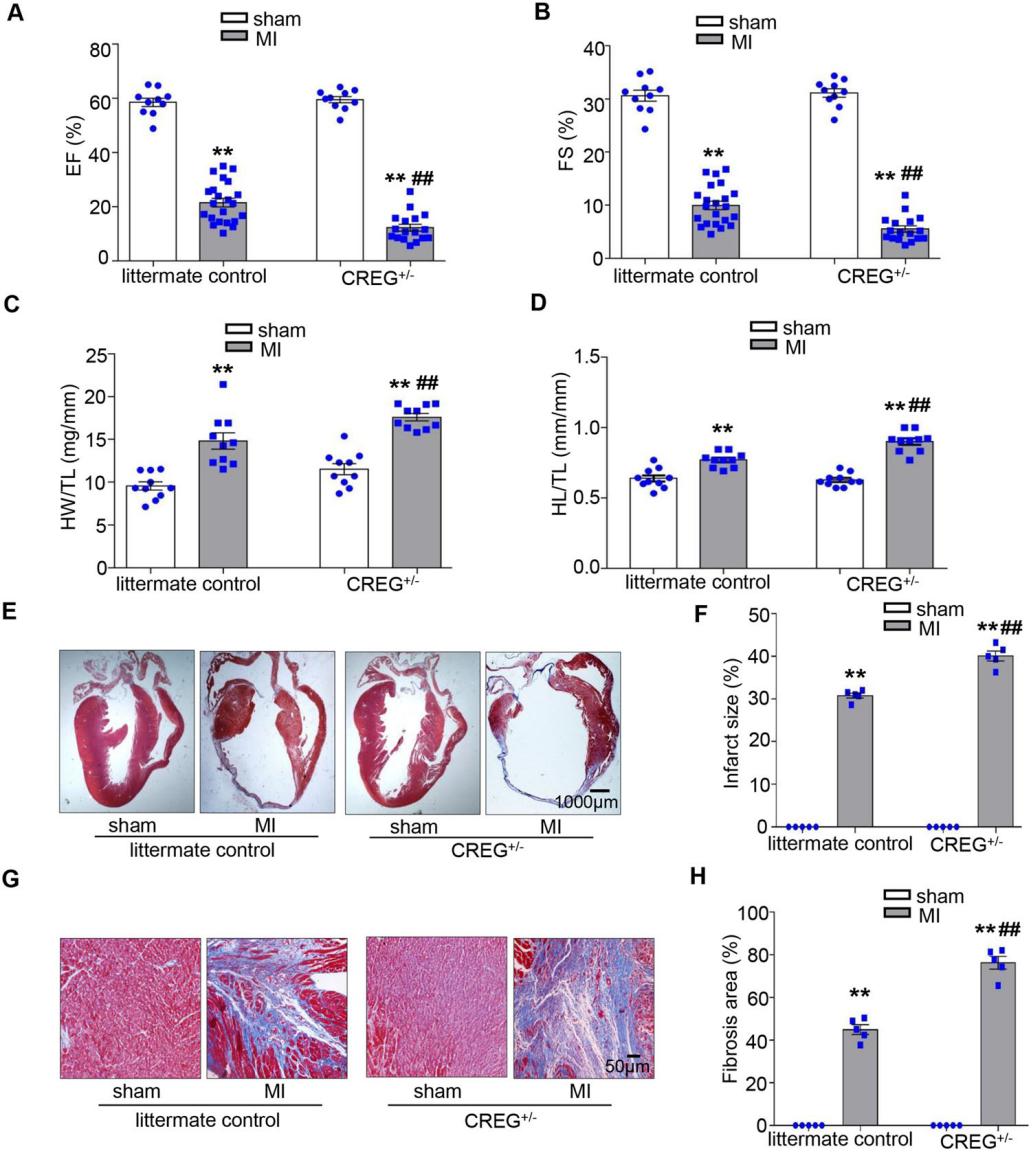
CREG deficiency exacerbated cardiac dysfunction and cardiac fibrosis on day 14 after MI. **A**, **B** EF% and FS% in CREG heterozygous mice (CREG^+/−^) mice and littermate control mice on post-MI day 14 (sham group, *N* = 10; MI group of littermate control mice, *N* = 22; and MI group of CREG^+/−^ mice, *N* = 17). **C**, **D** The heart weight-to-tibial length (HW/TL) ratio and heart length-to-tibial length (HL/TL) ratio in CREG^+/−^ mice and littermate control mice on post-MI day 14 (*N* = 10 per group). **E**, **F** Masson trichrome staining in CREG^+/−^ mice and littermate control mice on post-MI day 14 (*N* = 5 per group). **G**, **H** Fibrosis in border zone tissue in CREG^+/−^ mice and littermate control mice on post-MI day 14 (*N* = 5 per group). **P < 0.01 vs. sham group; ^##^*P* < 0.01 vs. littermate control-MI group.

To clarify the role of CREG in the development of post-MI heart failure, we evaluated cardiac function in CREG^+/−^ mice on post-MI days 3, 7, and 14. CREG mRNA and protein expression in the heart of CREG^+/−^ mice and controls were examined using real-time PCR and western blotting. CREG mRNA and protein expressions were significantly decreased in CREG^+/−^ mice compared with those in controls (*P* < 0.01, Fig. S1A–C). Compared with the sham group, EF% and FS% were significantly decreased in the MI group of CREG^+/−^ and controls on post-MI days 3 and 7 (*P* < 0.01, Fig. S1D, E), however, there was no difference in the MI group of CREG^+/−^ and littermate controls mice (Fig. S1D, E). Interestingly, compared with controls, EF% and FS% were significantly worsened in CREG^+/−^ mice on post-MI day 14 (EF: 12.24 ± 1.27% vs. 21.53 ± 1.60%, respectively; FS: 5.52 ± 0.60% vs. 9.96 ± 0.79%, respectively; *P* < 0.01, Fig. 2A, B). In addition, left ventricle internal diameter during diastole (LVIDd) and left ventricle internal diameter during systole (LVIDs) were increased in the MI group of CREG^+/−^ mice compared with that in the MI group of controls (LVIDd: 5.42 ± 0.16 mm vs. 5.10 ± 0.10 mm; LVIDs: 5.12 ± 0.15 mm vs. 4.60 ± 0.11 mm; *P* < 0.01, Table S1), besides, left ventricle posterior wall diameter during diastole (LVPWd) and left ventricle posterior diameter wall during systole (LVPWs) were decreased in CREG^+/−^ mice on post-MI day 14 (*P* < 0.01, Table S1).

### CREG deficiency aggravated myocardial fibrosis after MI

We investigated whether the deteriorated cardiac function in CREG^+/−^ mice was due, in part, to remodeling of the left ventricle by measuring the ratio of heart weight-to-tibial length (HW/TL) and the ratio of heart length-to-tibial length (HL/TL). HW/TL and HL/TL were increased in the MI group of CREG^+/−^ mice and controls compared with those in the sham group, and in the MI group of CREG^+/−^ mice compared with that in the MI group of controls (*P* < 0.01, Fig. 2C, D).

Masson’s trichrome staining was used to examine scar formation and fibrosis in CREG^+/−^ and controls following MI. There was no difference in the infarct size in the MI group of CREG^+/−^ mice and controls on post-MI days 3 and 7 (Fig. S1F, G). Notably, the infarct size in the MI group of CREG^+/−^ mice was significantly larger than that in the MI group of controls on post-MI day 14 (40.07 ± 1.16% vs. 30.79 ± 0.58%, respectively; *P* < 0.01, Fig. 2E, F). Compared with the sham group, fibrosis in the border zone of the myocardium was significantly increased in the MI group of CREG^+/−^ mice and controls on post-MI days 3 and 7 (*P* < 0.01, Fig. S1H, I). Furthermore, there was a significant increase in fibrosis in the border zone of CREG^+/−^ mice compared with that in controls on post-MI day 14 (76.18 ± 2.99% vs. 44.89 ± 2.29%, respectively; *P* < 0.01, Fig. 2G, H).

### CREG deletion in cardiomyocyte did not impair cardiac function following MI

To clarify the role of CREG in cardiomyocyte on cardiac function following MI, CREG-CKO mice were generated and identified (Fig. S2A, B), and the mRNA and protein expressions of CREG in the myocardium of CREG-CKO mice and flox/flox mice were examined. Compared with flox/flox mice, the mRNA and protein levels of CREG were significantly decreased in CREG-CKO mice (*P* < 0.01, Fig. S2C–E). Subsequently, CREG-CKO mice and flox/flox mice were performed for the MI model, and the cardiac function (EF% and FS%) on post-MI day 14 was evaluated. The results demonstrated that EF% and FS% were significantly decreased in the MI group of CREG-CKO and flox/flox mice compared with that in the sham group (*P* < 0.01, Fig. S2F, G). However, no significant difference was observed between CREG-CKO and flox/flox mice on post-MI day 14 (*P* > 0.05, Fig. S2F, G). These results indicated that CREG in cardiomyocytes might not play a vital role in the development of MI.

### Exogenous CREG recombinant protein ameliorated cardiac dysfunction after MI

**Fig. 3:**
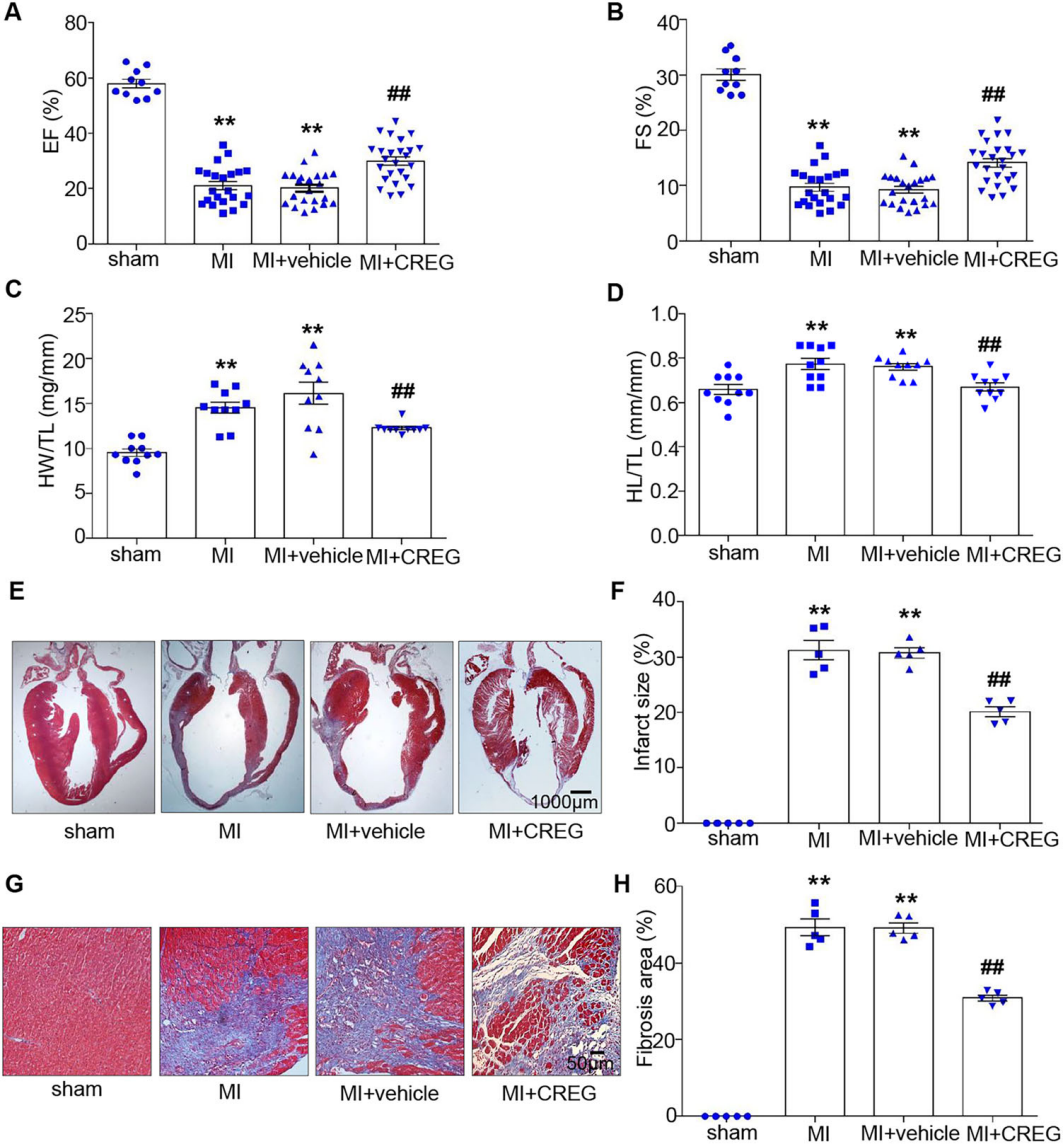
CREG protein alleviated cardiac dysfunction and cardiac fibrosis on day 14 after MI. **A**, **B** EF% and FS% in C57BL/6J mice with overexpression of exogenous CREG on post-MI day 14 (*N* = 10 in sham group; *N* = 22 in MI and MI + vehicle groups; *N* = 25 in MI + CREG group). **C**, **D** The heart weight-to-tibial length (HW/TL) ratio and heart length-to-tibial length (HL/TL) ratio in C57BL/6J mice with overexpression of exogenous CREG on post-MI day 14 (*N* = 10 per group). **E**, **F** Masson trichrome staining in C57BL/6J mice with exogenous CREG recombinant protein on post-MI day 14 (*N* = 5 per group). **G**, **H** Fibrosis in the border zone tissue in C57BL/6J mice with overexpression of exogenous CREG on post-MI day 14 (*N* = 5 per group). **P < 0.01 vs. sham group; ^##^*P* < 0.01 vs. MI + vehicle group.

Cardiac function was evaluated on post-MI day 14 to determine whether CREG protein could improve it. Interestingly, compared with those in MI + vehicle group, EF% and FS% were significantly improved in MI + CREG group (EF: 29.96 ± 1.51% vs. 20.20 ± 1.27%, respectively; FS: 14.14 ± 0.77% vs. 9.27 ± 0.61%, respectively; *P* < 0.01, Fig. 3A, B). In addition, LVIDs were reduced in MI + CREG group compared with that in MI + vehicle group on post-MI day 14 (4.11 ± 0.10 mm vs. 4.59 ± 0.11 mm, *P* < 0.01, Table S2).

### Exogenous CREG protein alleviated myocardial fibrosis after MI

We investigated whether the enhanced cardiac function observed with exogenous CREG treatment was related to reducing remodeling. Compared with MI + vehicle group, CREG protein significantly reduced HW/TL and HL/TL in MI + CREG group on post-MI 14 days (*P* < 0.01, Fig. 3C, D).

The infarct sizes in MI and MI + vehicle group were significantly increased compared with those in the sham group (*P* < 0.01, Fig. 3E, F); however, CREG protein significantly reduced the infarct size (20.14 ± 0.89% vs. 30.76 ± 0.93%, respectively; *P* < 0.01, Fig. 3E, F). Furthermore, there was a substantial decrease in fibrosis in the border zone of the MI + CREG group compared with that in the MI + vehicle group (30.84 ± 0.77% vs. 49.12 ± 1.33%, respectively; *P* < 0.01, Fig. 3G, H). These results revealed that CREG protein could improve cardiac function and alleviate cardiac fibrosis following MI.

### CREG deficiency aggravated cardiac myofibroblast activation in the border zone of the myocardium after MI

Post-MI cardiac fibrosis is mainly influenced by cardiomyocyte apoptosis and cardiac myofibroblast activation^5,23^. Dramatic apoptosis was observed in the border zone of CREG^+/−^ and controls on post-MI days 3, 7, and 14; however, no significant difference was observed between CREG^+/−^ and controls (Fig. S3A–C). Therefore, the result indicated that cardiomyocyte apoptosis might not play a key role in post-MI CREG-mediated myocardial fibrosis.

**Fig. 4:**
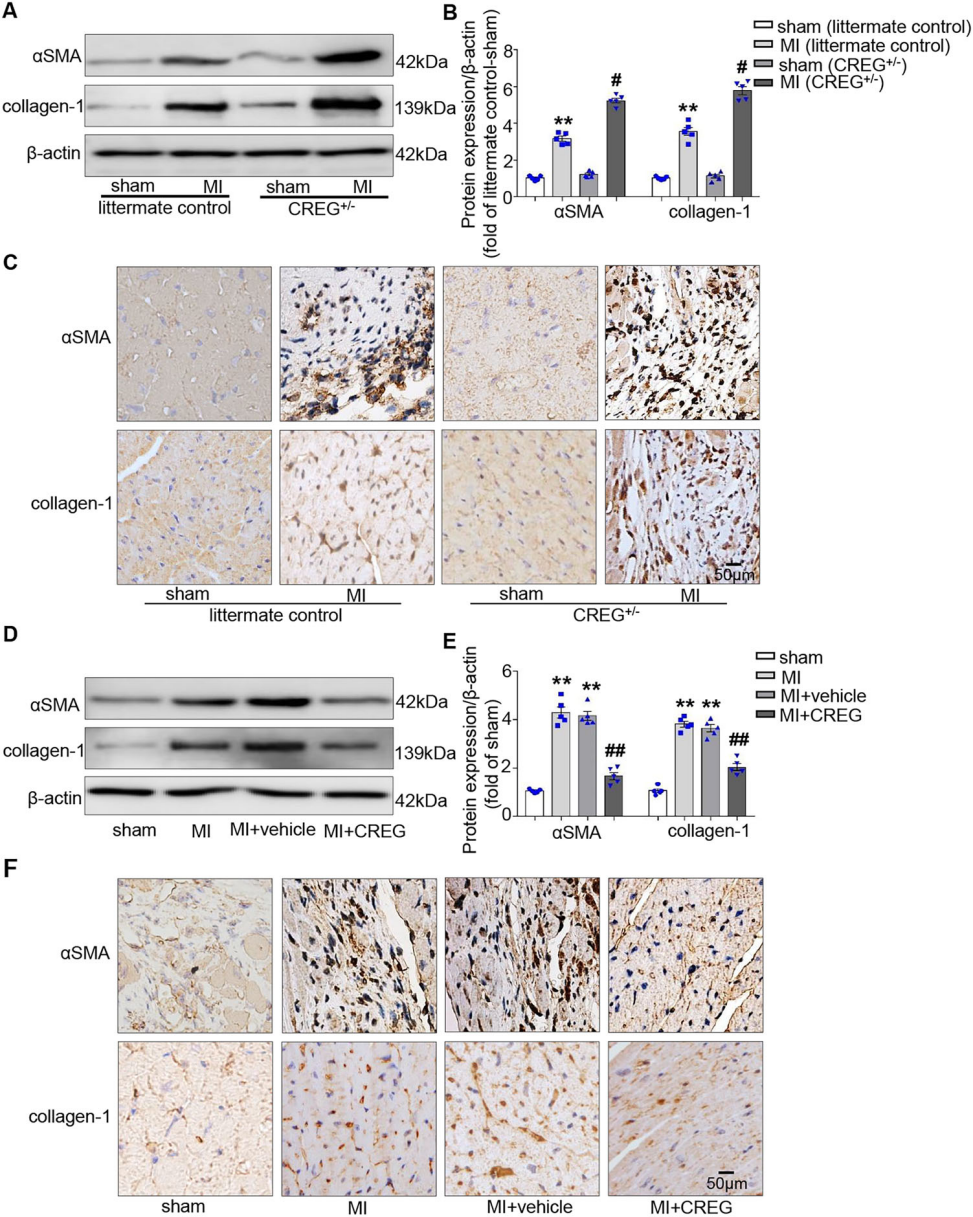
CREG recombinant protein inhibited cardiac myofibroblast activation in the border zone of the myocardium after MI. **A**, **B** Western blotting analysis of αSMA and collagen-1 expression in the border zone of the myocardium in CREG heterozygous mice (CREG^+/−^) on post-MI day 14 (*N* = 5 per group). **C** Immunohistochemical analysis of αSMA and collagen-1 expression in the border zone of the myocardium in CREG^+/−^ mice on post-MI day 14 (*N* = 3 per group). **D**, **E** Western blotting of αSMA and collagen-1 expression in the border zone of the myocardium in C57BL/6J mice with exogenous CREG recombinant protein (rCREG) on post-MI day 14 (*N* = 5 per group). **F** Immunohistochemical analysis of αSMA and collagen-1 expression in the border zone of the myocardium in C57BL/6J mice with rCREG on post-MI day 14 (*N* = 3 per group). **P < 0.01 vs. sham group; ^#^*P* < 0.05 vs. littermate control-MI, ^##^*P* < 0.01 vs. MI + vehicle group.

Cardiac myofibroblast activation is a key step in cardiac remodeling, and αSMA and collagen-1 are the commonest markers of cardiac myofibroblast activation^24^. Therefore, we examined the expressions of αSMA and collagen-1 in the border zone of CREG^+/−^ mice on post-MI day 14. αSMA and collagen-1 protein levels were significantly increased in the MI group of CREG^+/−^ mice and controls compared with those in the sham group (*P* < 0.05, Fig. 4A, B). The immunohistochemistry results were consistent with the western blotting results (Fig. 4C). Besides, compared with the MI group of controls, αSMA and collagen-1 protein expressions were significantly increased in the MI group of CREG^+/−^ mice (*P* < 0.05, Fig. 4A–C). Furthermore, the proliferation of cardiac fibroblasts on post-MI day 14 in CREG^+/−^ mice and littermate control mice was evaluated using flow cytometry. The percentage of Lin^−^GP38^+^ cells in the MI group of CREG^+/−^ mice was significantly higher compared with that in the MI group of littermate control mice (Fig. S3D, E). These results indicated that CREG deficiency enhanced cardiac myofibroblast activation in the border zone of the myocardium following MI.

### CREG protein alleviated cardiac myofibroblast activation in the border zone of the myocardium after MI

To further clarify the role of CREG in cardiac myofibroblast activation, the expressions of αSMA and collagen-1 were examined in the border zone of C57BL/6J mice using CREG exogenous protein on post-MI day 14. These protein levels were significantly reduced in MI + CREG group compared with those in MI + vehicle group (*P* < 0.01, Fig. 4D, E). Furthermore, immunohistochemical analysis revealed that CREG protein greatly decreased αSMA and collagen-1 levels following MI (Fig. 4F). These results demonstrated that CREG protein could play a pivotal role in cardiac myofibroblast activation in vivo.

### Knockdown of CREG aggravated cardiac myofibroblast activation in vitro

**Fig. 5:**
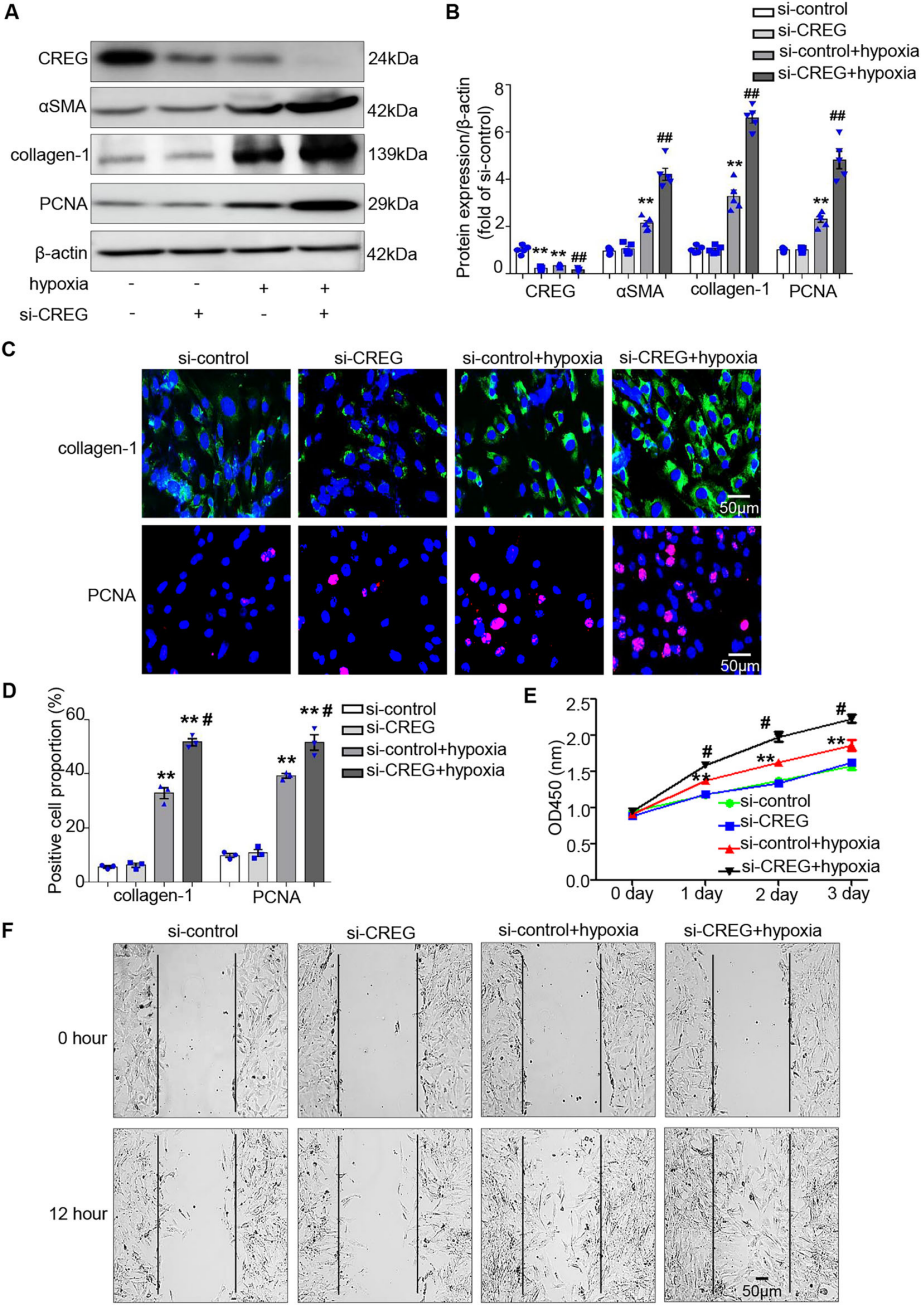
Knockdown of CREG enhanced the phenotypic switching of cardiac fibroblasts in vitro. **A**, **B** The effects of CREG siRNA (si-CREG) on the expression of αSMA and collagen-1 induced by hypoxia as determined by western blotting (*N* = 5 per group). **C**, **D** The effects of si-CREG on the expression of collagen-1 and PCNA induced by hypoxia as determined by immunofluorescence staining (*N* = 3 per group). Green indicated collagen-1 expression, red indicated PCNA expression, and blue indicated nuclei. **E** The effects of si-CREG on cell proliferation were determined by CCK8 assays (*N* = 5 per group). **F** The effects of si-CREG on cell migration were determined by wound healing assays (*N* = 3 per group). **P < 0.01 vs. si-control group; ^##^*P* < 0.01, ^#^*P* < 0.05 vs. si-control+hypoxia group.

Similar to the in vivo findings, hypoxia dramatically reduced CREG protein expression compared with the control group (*P* < 0.01, Fig. 5A, B). In addition, both αSMA and collagen-1 expressions were upregulated in the hypoxia group (*P* < 0.01, Fig. 5A–D). To further determine whether CREG knockdown enhanced hypoxia-induced cardiac myofibroblast activation, cardiac fibroblasts were transfected with si-CREG and subjected to hypoxia stimulation. Compared with the si-control group, there were no obvious changes in the expressions of αSMA, collagen-1, and PCNA and migration and proliferation of cardiac fibroblasts in the si-CREG group (*P* > 0.05, Fig. 5A–F). However, CREG knockdown enhanced the expressions of αSMA and collagen-1 induced by hypoxia (*P* < 0.01, Fig. 5A–D). In addition, CREG deficiency greatly increased the proliferation and migration of cardiac fibroblasts induced by hypoxia (*P* < 0.05, Fig. 5A–F).

### CREG protein inhibited hypoxia-induced cardiac myofibroblast activation in vitro

**Fig. 6:**
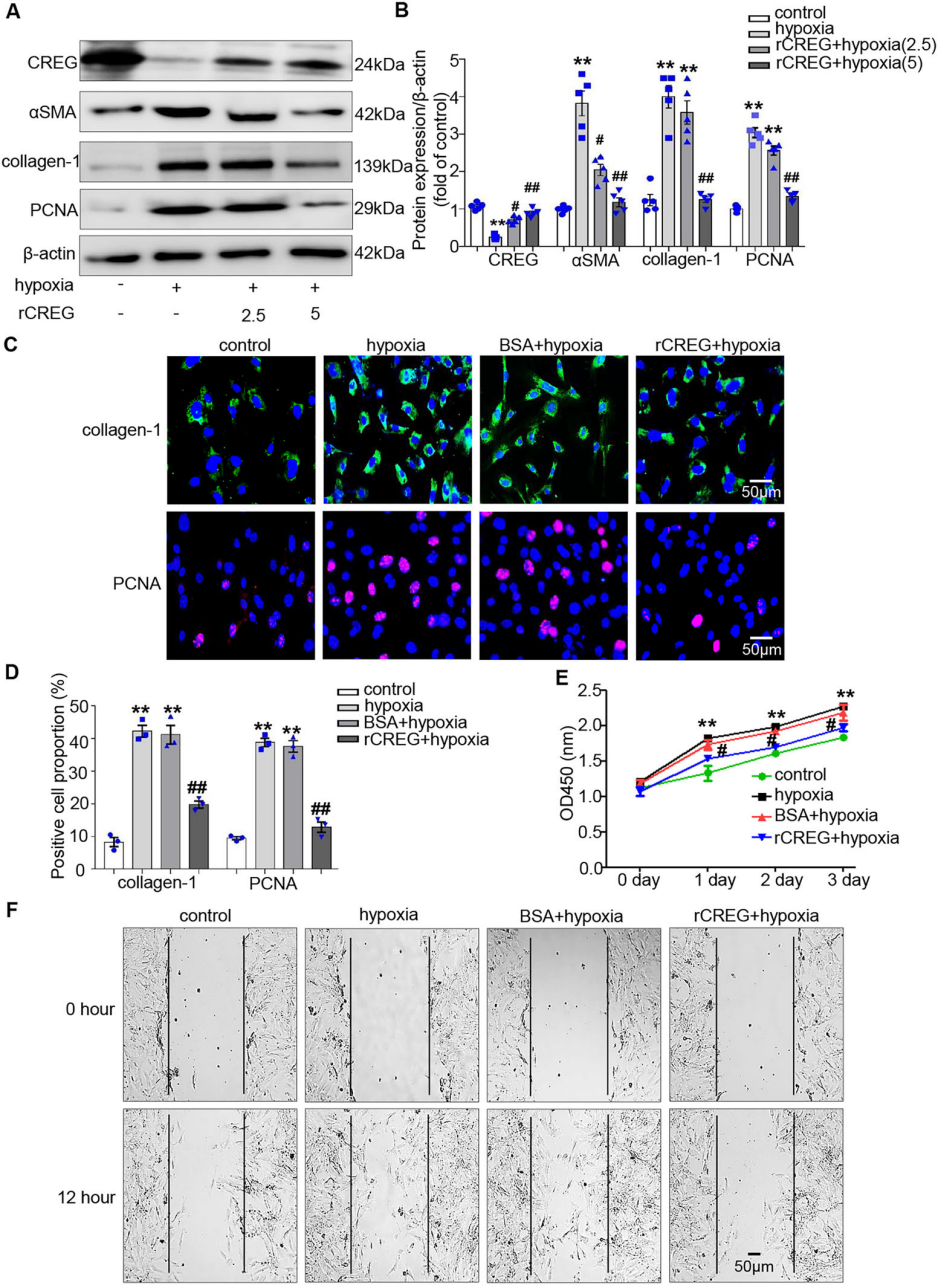
CREG protein inhibited the phenotypic switching of cardiac fibroblasts in vitro. **A**, **B** The effects of CREG recombinant protein (rCREG) on the expression of αSMA and collagen-1 induced by hypoxia as determined by western blotting (*N* = 5 per group). **C**, **D** The effects of rCREG on the expression of collagen-1 and PCNA induced by hypoxia as determined by immunofluorescence staining (*N* = 3 per group). Green indicated collagen-1 expression, red indicated PCNA expression, and blue indicated nuclei. **E** The effects of rCREG on cell proliferation were determined by CCK8 assays (*N* = 5 per group). **F** The effects of rCREG on cell migration was determined by wound healing assays (*N* = 3 per group). **P < 0.01 vs. control group; ^##^*P* < 0.01, ^#^*P* < 0.05 vs. BSA + hypoxia group.

To determine whether CREG protein could reverse hypoxia-induced cardiac myofibroblast activation, the cells were pretreated with different concentrations of CREG protein (rCREG) and subjected to hypoxia, CREG mRNA and protein expressions were examined. There was no difference in mRNA expression between rCREG and bovine serum albumin (BSA) groups (Fig. S4A), however, CREG protein was significantly increased in the rCREG group compared with the BSA group (*P* < 0.01, Fig. S4B, C). Interestingly, CREG protein inhibited the expression of αSMA and collagen-1 induced by hypoxia (*P* < 0.01, Fig. 6A–D). Subsequently, further evaluations revealed that CREG protein could greatly inhibit the proliferation and migration of cardiac fibroblasts induced by hypoxia (*P* < 0.05, Fig. 6A–F).

### The effect of CREG on cardiac myofibroblast activation was independent of IGF2R

Previous studies have demonstrated that IGF2R is a receptor of CREG protein^25^. The effects of CREG on phenotype transformation in cardiac fibroblasts after hypoxia were independent of IGF2R in this study. The expression levels of CREG mRNA and protein were not altered in the IGF2R knockdown group (Fig. S4D–F). In addition, the effects of CREG on αSMA expression under hypoxia were not influenced by IGF2R knockdown (Fig. S4G, H). Therefore, an unknown mechanism other than IGF2R may mediate the role of CREG on cardiac myofibroblast activation following MI.

### CDC42 protein expression was upregulated in the myocardium of CREG^+/−^ mice

**Fig. 7:**
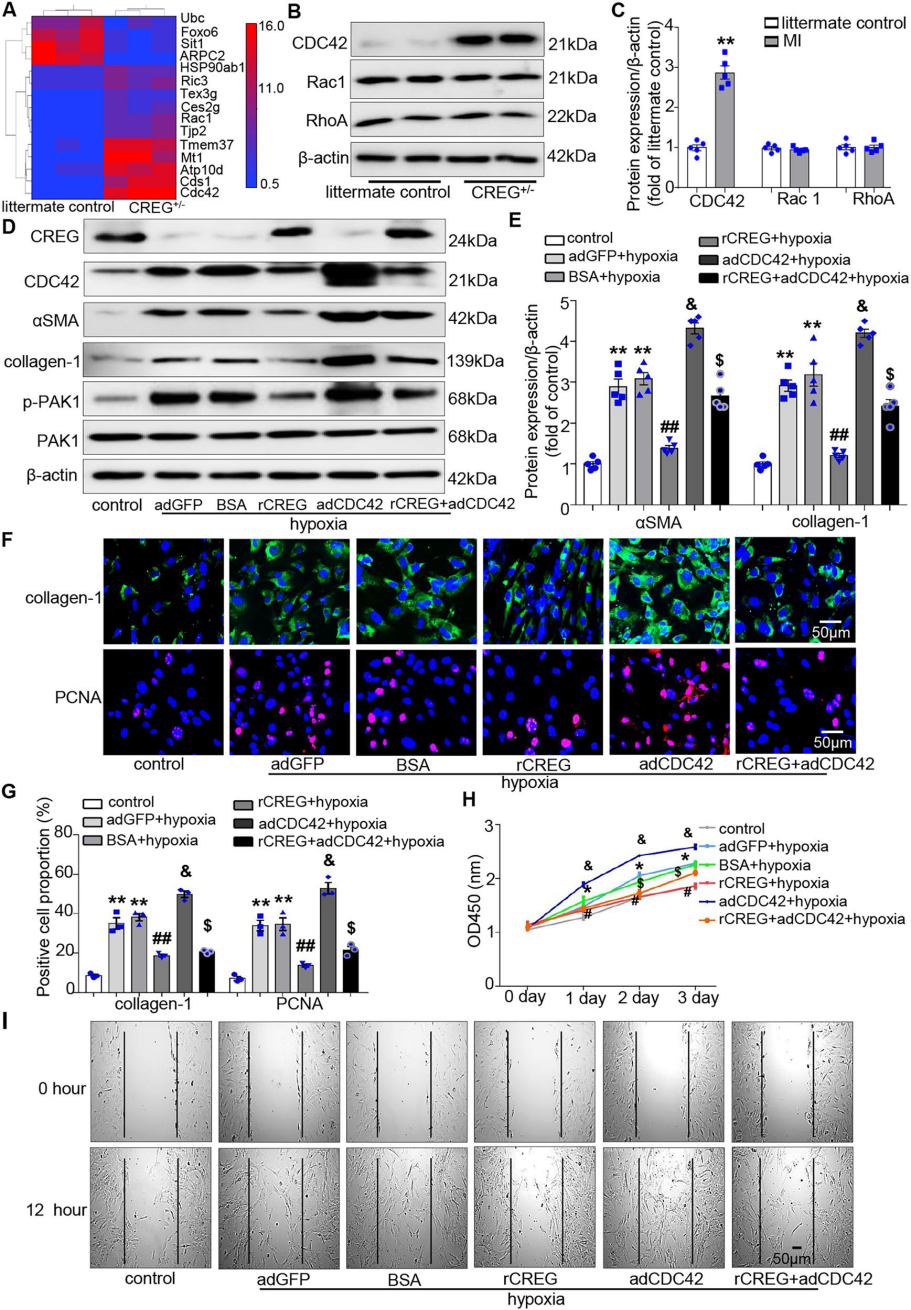
CREG protein inhibited hypoxia-induced cardiac myofibroblast activation by regulating CDC42 expression. **A** Mass spectrometry in the myocardium of CREG^+/−^ mice and littermate control mice (*N* = 3 per group). **B**, **C** Western blotting of CDC42, Rac1, and RhoA expression in the myocardium of CREG^+/−^ mice (*N* = 5 per group). **D**, **E** The effects of CREG recombinant protein (rCREG) and CDC42 overexpressed-adenovirus (adCDC42) on the expression of αSMA and collagen-1 induced by hypoxia as determined by western blotting (*N* = 5 per group). **F**, **G** The effects of rCREG and adCDC42 on the expression of collagen-1 and PCNA induced by hypoxia as determined by immunofluorescence staining (*N* = 3 per group). Green indicated collagen-1 expression, red indicated PCNA expression, and blue indicated nuclei. **H** CCK8 assays were used to evaluate the effects of rCREG and adCDC42 on cell proliferation (*N* = 5 per group). **I** The effects of rCREG and adCDC42 on cell migration were determined by wound healing assays (*N* = 3 per group). **P < 0.01, ^*^P < 0.05 vs. littermate control or control group; ^##^*P* < 0.01, ^#^*P* < 0.05 vs. BSA + hypoxia group; ^&^*P* < 0.05 vs. adGFP+hypoxia; ^$^*P* < 0.05 vs. adCDC42+ hypoxia group.

Mass spectrometry revealed that 15 proteins were differentially expressed in the myocardium of the CREG^**+/−**^ group compared with the controls (Fig. 7A). Interestingly, two members (CDC42 and Rac1) of the Rho GTPases family were increased in CREG^+/−^ mice. Previous studies have demonstrated that Rho GTPase plays an important role in fibrosis^26,27^; therefore, the expression of Rho GTPase members was verified. CDC42 expression was significantly increased in the myocardium of CREG^+/−^ mice (*P* < 0.01, Fig. 7B, C). However, Rac1 and RhoA expression was unchanged in the myocardium of CREG^+/−^ mice (*P* > 0.05, Fig. 7B, C). Therefore, CDC42 might be a key molecule in the process of CREG-mediated cardiac myofibroblast activation.

### CREG protein inhibited cardiac myofibroblast activation by regulating CDC42 expression in vitro

PAK1 is a direct target of CDC42 in the heart^28^; therefore, to clarify the roles of CDC42 in CREG-mediating cardiac myofibroblast activation, the expression levels of CDC42 and PAK1 were firstly examined in hypoxia-induced cardiac fibroblasts. Hypoxia increased the expressions of CDC42 and phospho-PAK1 in vitro (*P* < 0.01, Fig. 7D, E). Furthermore, compared with the adGFP + hypoxia group, CDC42 overexpression could increase the expressions of αSMA and collagen-1 and proliferation and migration of cardiac fibroblasts induced by hypoxia (*P* < 0.05, Fig. 7D–I).

To clarify the role of CDC42 in mediating the effects of CREG in cardiac myofibroblast activation, adCDC42 was delivered into cells with CREG recombinant protein stimulation. Interestingly, αSMA, collagen-1, and proliferation and migration of cardiac fibroblasts were increased in the rCREG + adCDC42 + hypoxia group compared with those in the rCREG+hypoxia group (*P* < 0.05, Fig. 7D–I). Conversely, αSMA and collagen-1 expression and proliferation and migration of cardiac fibroblasts were greatly decreased in the rCREG + adCDC42 + hypoxia group compared with those in the adCDC42+hypoxia group (*P* < 0.05, Fig. 7D–I). The above results indicated that CREG protein could inhibit the phenotypic transformation of cardiac fibroblasts induced by hypoxia via modulation of CDC42 expression.

### CREG inhibited CDC42 protein expression and activity in cardiac fibroblasts

**Fig. 8:**
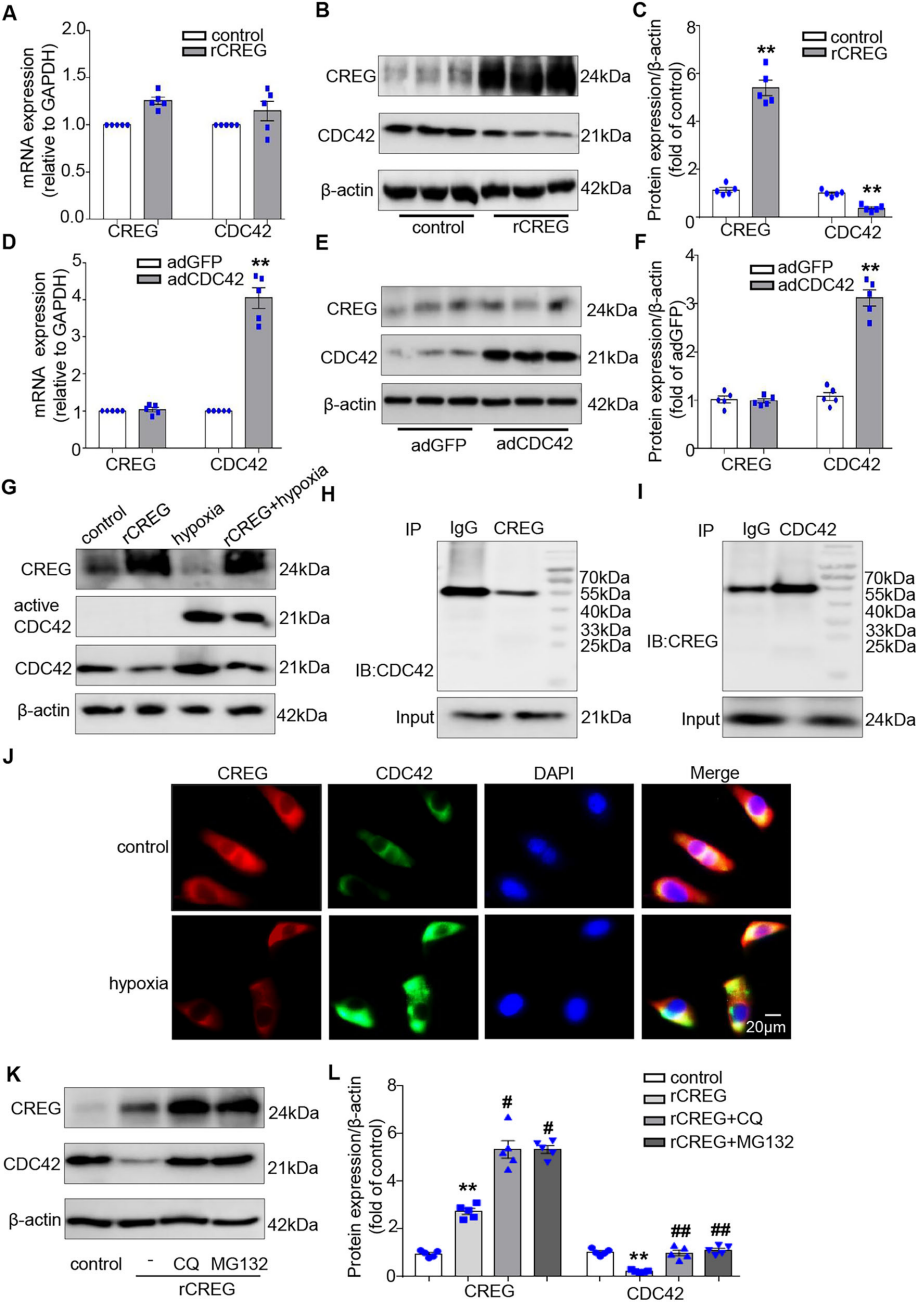
CREG protein inhibited CDC42 expression and activity in vitro. **A** Real-time PCR of *CDC42* mRNA expression in cardiac fibroblasts with CREG recombinant protein (rCREG, *N* = 5 per group). **B**, **C** Western blotting of CDC42 protein expression in cardiac fibroblasts with rCREG (*N* = 5 per group). **D** Real-time PCR of *CREG* mRNA expression in cardiac fibroblasts with CDC42 overexpressed-adenovirus (adCDC42, *N* = 5 per group). **E**, **F** Western blotting of CREG protein expression in cardiac fibroblasts with adCDC42 (*N* = 5 per group). **G** Pull-down assay to determine if the effects of rCREG on the CDC42 activity in mouse cardiac fibroblasts under hypoxia (*N* = 3 per group). **H**, **I** Immunoprecipitation of CDC42 with CREG or CREG with CDC42 in mouse cardiac fibroblasts (*N* = 3 per group). **J** Immunofluorescence staining for CREG and CDC42 in cardiac fibroblasts (*N* = 3 per group). **K**, **L** Western blotting of CREG and CDC42 expression in chloroquine-treated or MG132-treated cardiac fibroblasts. Red indicated CREG expression, green indicated CDC42 expression, blue indicated nuclei, and yellow indicated the co-location of CREG with CDC42 (*N* = 5 per group). **P < 0.01 vs. control or adGFP group; ^##^*P* < 0.01, ^#^*P* < 0.05 vs. rCREG group.

To clarify the relationship between CREG and CDC42, mRNA and protein levels of CREG and CDC42 were examined in cardiac fibroblasts with CREG recombinant protein or CDC42 overexpressed adenovirus. CDC42 protein expression was significantly decreased in the rCREG group, although CDC42 mRNA expression was unchanged in this group (Fig. 8A–C). However, CREG mRNA and protein levels were not influenced by CDC42 overexpression (Fig. 8D–F). In addition, the effects of CREG on CDC42 activity were examined under normoxia and hypoxia. CREG protein could inhibit CDC42 activity induced by hypoxia (Fig. 8G), although CDC42 activity was not affected by CREG protein under normoxia (Fig. S5A). Furthermore, the interaction and co-location between CREG and CDC42 were also evaluated. There was no direct interaction between CREG protein and CDC42 protein (Fig. 8H, I); however, CREG and CDC42 could be co-located in cardiac fibroblasts under normoxia and hypoxia (Fig. 8J).

To further examine the role of CREG in CDC42 protein degradation, cells were treated with chloroquine (CQ, lysosome inhibitor, 10 μM) or MG132 (proteasome inhibitor, 10 μM) for 24 h. CQ and MG132 dramatically reversed CDC42 expression inhibited by CREG protein (*P* < 0.01, Fig. 8K, L). These results indicated that CREG protein inhibited CDC42 expression via the lysosome and proteasome pathways.

### CREG recombinant protein decreased CDC42 expression in the border zone of the myocardium after MI

To clarify the effects of MI on CDC42 expression in vivo, we evaluated the protein expressions of CDC42 and phospho-PAK1 in the border zone of the myocardium of C57BL/6J mice on post-MI day 14. CDC42 and phospho-PAK1 levels were increased in MI and MI + vehicle groups compared with those in the sham group (*P* < 0.01, Fig. S5B, C). Notably, CREG protein inhibited the expressions of CDC42 and phospho-PAK1 in the border zone of the myocardium in vivo (Fig. S5B, C).

## Discussion

Cardiac fibrosis plays important role in post-MI cardiac repair. Several factors are implicated in MI-induced cardiac fibrosis including apoptosis^23^, inflammation^29^, and cardiac fibroblast activation^5^. Cardiac fibroblasts are the key cellular components of post-MI ventricular fibrosis. Following MI, cardiac fibroblasts differentiate into myofibroblasts, which cause abnormal myocardial stiffness and impairment of cardiac function. Therefore, clarification of the molecular mechanisms that regulate cardiac myofibroblast activation could provide new targets for the prevention and treatment of post-MI cardiac fibrosis.

CREG is an important glycoprotein that regulates the homeostasis of tissues and cells. It is a protective factor against aging or stress-induced myocardial damage^17,18^. Transplantation of CREG-modified embryonic stem cells (ESCs) improved post-MI cardiac function in mice, while CREG overexpression with adenovirus inhibits ESC apoptosis and enhances their differentiation into cardiomyocytes in vitro^15^. Furthermore, CREG-overexpressing bone mesenchymal stem cell transplantation protects against MI in rats by promoting vascular endothelial growth factor-induced anti-apoptotic effects and angiogenesis^16^. However, the effects and mechanisms of CREG in post-MI cardiac myofibroblast activation remain unknown.

In our study, CREG protein expression was downregulated in the border zone of myocardium on post-MI days 7 and 14 along with cardiac fibroblast activation, which indicated that CREG might participate in the development of MI by affecting cardiac fibroblast activation. In vivo loss-of-function and gain-of-function experiments were performed to clarify the roles of CREG in post-MI cardiac myofibroblast activation. In vivo, CREG deficiency impaired cardiac function and induced severe myocardial fibrosis and cardiac myofibroblast activation following MI. Conversely, CREG protein significantly reversed the post-MI cardiac damage and inhibited cardiac myofibroblast activation. In vitro, CREG protein inhibited the phenotypic switching of cardiac fibroblasts by blocking αSMA and collagen-1 expression and suppressing the proliferation and migration of cardiac fibroblasts induced by hypoxia. These results suggest that CREG could be a new potential target to prevent cardiac myofibroblast activation and cardiac fibrosis after MI.

CDC42 could mediate the effects of CREG in cardiac myofibroblast activation in vitro. CDC42 is a member of the Rho GTPase family and plays important roles in response to physiological and pathological stimulation^30,31^. When cardiomyocytes are subjected to pathological stimulation, CDC42 becomes activated and binds to PAK1, which in turn induces the activation of PAK1^27^. Activated PAK1 plays vital roles in several cellular biological processes, such as growth, survival, and death. The inhibition of CDC42 decreases myocardial fibrosis and hypertrophy in salt-sensitive hypertension^32^. In addition, increases in CDC42 and PAK1 levels induced by miR-30c could promote diabetes-related cardiac hypertrophy^33^. Conversely, several studies have reported that CDC42 and PAK1 play anti-hypertrophic roles. CDC42-knockout and PAK1-knockout mice develop greater cardiac hypertrophy compared with controls^26,34^, which demonstrates the contradictory roles of CDC42 and PAK1 in cardiac hypertrophy. However, the role of CDC42 and PAK1 in post-MI cardiac fibrosis was unknown.

In this study, CDC42 and activated PAK1 levels were increased in the border zone of the myocardium following MI, and CREG protein blocked these effects. In addition, CDC42 was a downstream effector of CREG, and its expression was inhibited by CREG protein via the lysosome and proteasome pathways. Therefore, CDC42 was a key molecule that mediates the effects of CREG in post-MI cardiac myofibroblast activation. PAK1 has been demonstrated to play important roles in cell growth by regulating the activation of extracellular signal-regulated protein kinase and mitogen-activated protein kinase pathways^22^. However, further studies are required to determine whether the above signaling pathways participate in the role of the CREG-CDC42-PAK1 axis in post-MI cardiac myofibroblast activation.

There were several limitations in our study. Firstly, both injury and rescue models do not offer CREG fibroblast-specific deletion or overexpression in vivo. Secondly, as an extracellular glycoprotein, CREG would be expected to signal through a surface receptor; however, the CREG receptor remains unclear. In future studies, fibroblast-specific conditional deletion or overexpression models and the direct receptor of CREG should be investigated.

In conclusion, we demonstrated the protective roles of CREG in post-MI cardiac myofibroblast activation via the inhibition of CDC42 protein expression and activity (Fig. S6). Our findings could provide a promising preventive and therapeutic target for mediating post-MI cardiac fibrosis and cardiac myofibroblast activation.

## Supplementary information

Suppmentary material
