# Supplementary material for: CREG ameliorates the phenotypic switching of cardiac fibroblasts after myocardial infarction via modulation of CDC42
Source: Cell Death Dis. 2021 Apr 6;12(4):355. doi: 10.1038/s41419-021-03623-w (PMC8024263; doi:10.1038/s41419-021-03623-w)
Supplement: Supplementary file 1 — Suppmentary material [file 41419_2021_3623_MOESM1_ESM.pdf]

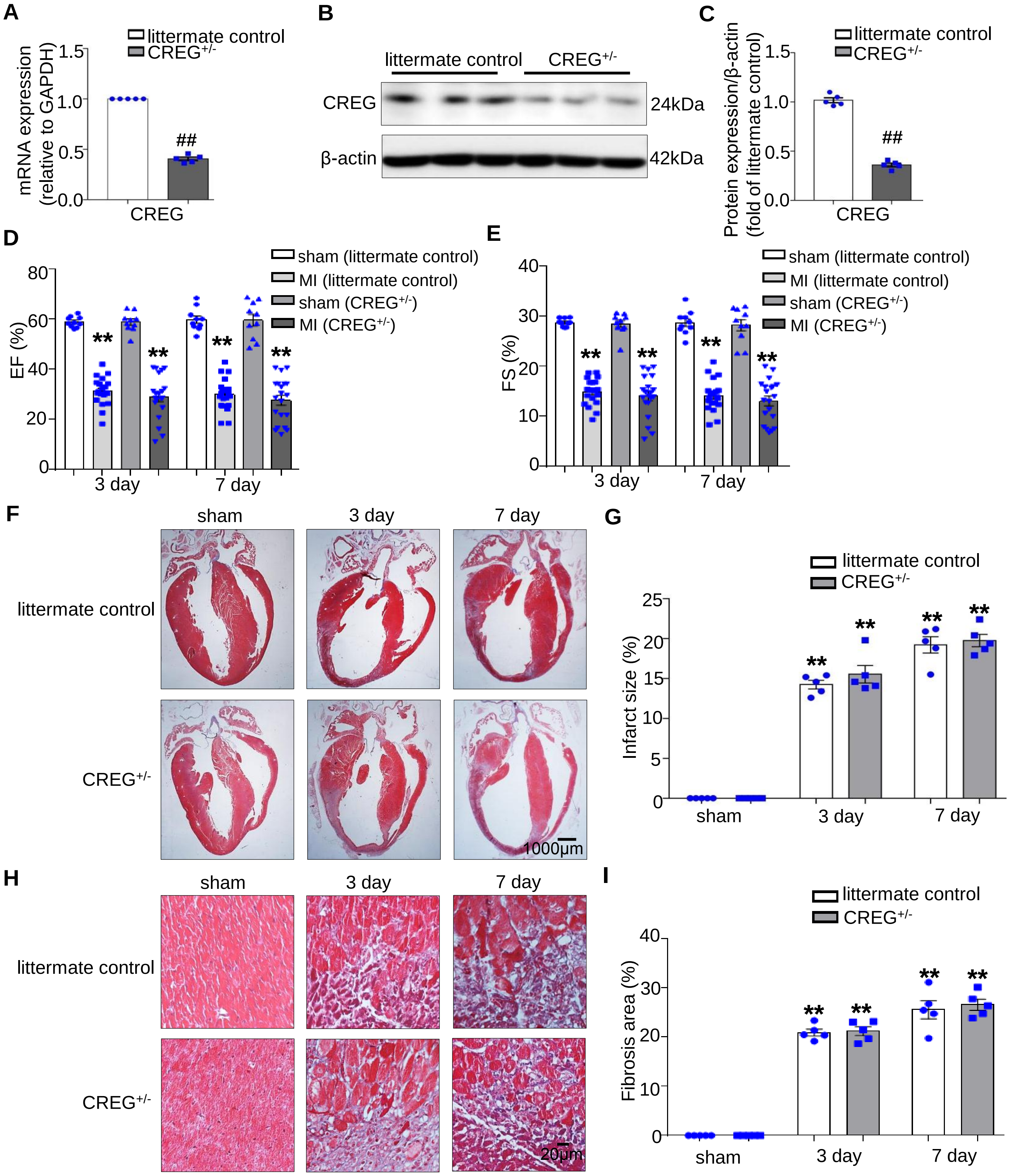

Figure S1. The cardiac function and fibrosis in CREG$^{+/-}$ mice and littermate control mice following MI.
A. *CREG* mRNA expression in the heart of CREG$^{+/-}$ mice and littermate control mice, N=5 each group. **B, C**. CREG protein expression in the heart of CREG$^{+/-}$ mice and littermate control mice, N=5 each group. **D, E**. EF% and FS% in CREG$^{+/-}$ mice and littermate control mice on days 3 and 7 after MI. N=10 for sham group, N=20 for MI group. **F, G**. Masson staining of CREG$^{+/-}$ mice and littermate control mice on days 3 and 7 after MI. N=5 each group. **H, I**. Fibrosis of border zone tissue in CREG$^{+/-}$ mice and littermate control mice on days 3 and 7 after MI. N=5 per group. **P<0.01 vs. sham group, ##P<0.01 vs. littermate control.

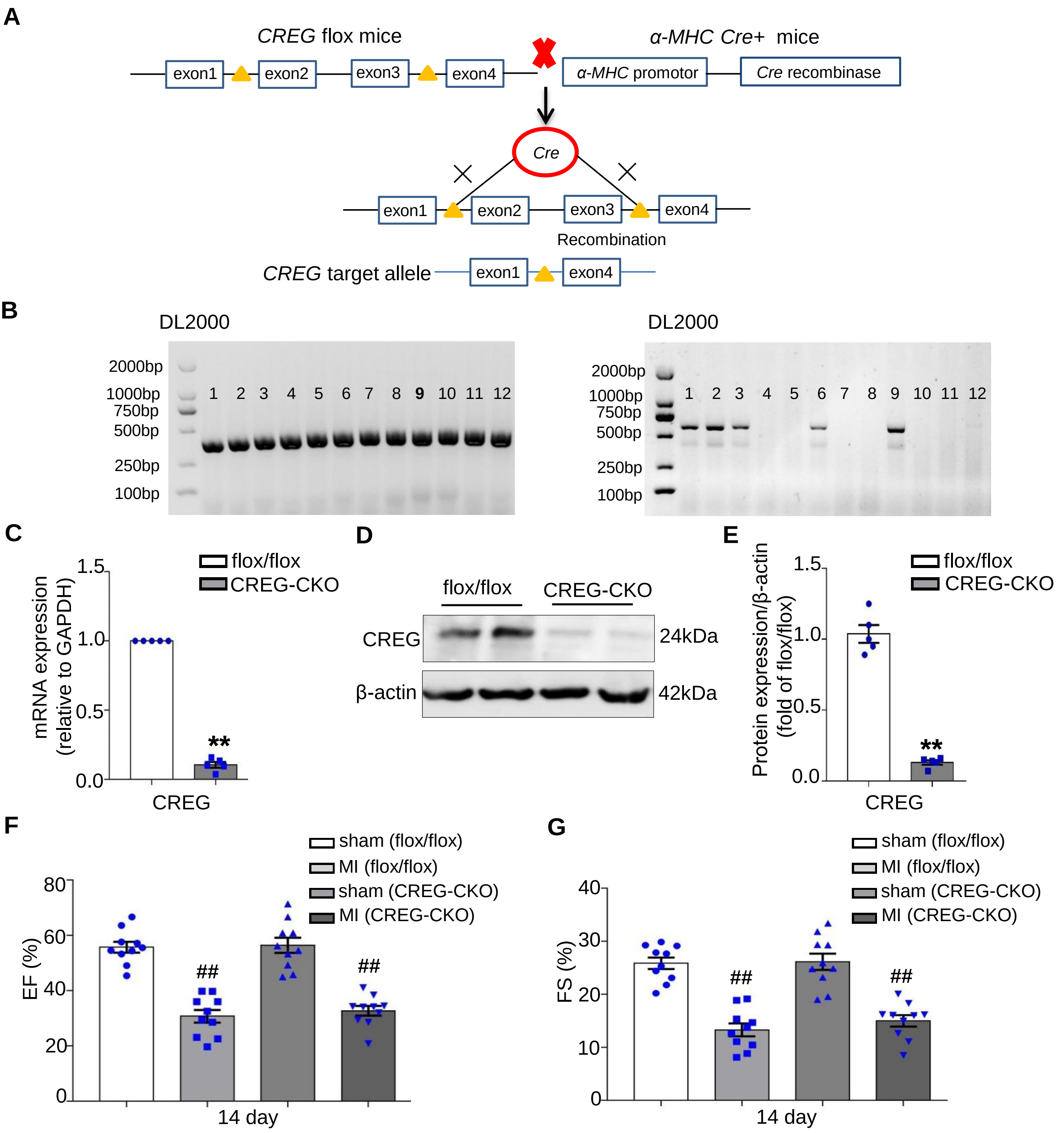

Figure S2. The role of CREG in cardiomyocytes on cardiac function on day 14 following MI

A. Construction of cardiomyocyte-specific CREG knockout mice (CREG-CKO). B. Genotyping of CREG-CKO mice and flox/flox mice. CREG-CKO mice: 1, 2, 3, 6 and 9; flox/flox mice: 4, 5, 7, 8, 10, 11 and 12. **C.** *CREG* mRNA expression in the myocardium of CREG-CKO and flox/flox mice, N=5 each group. **D, E.** CREG protein expression in the myocardium of CREG-CKO mice and flox/flox mice, N=5 each group. **F, G.** EF% and FS% of CREG-CKO mice and flox/flox mice on day 14 following MI. N=10 each group. **P<0.01 vs. flox/flox group; $^{\#\#}$P<0.01 vs. sham group.

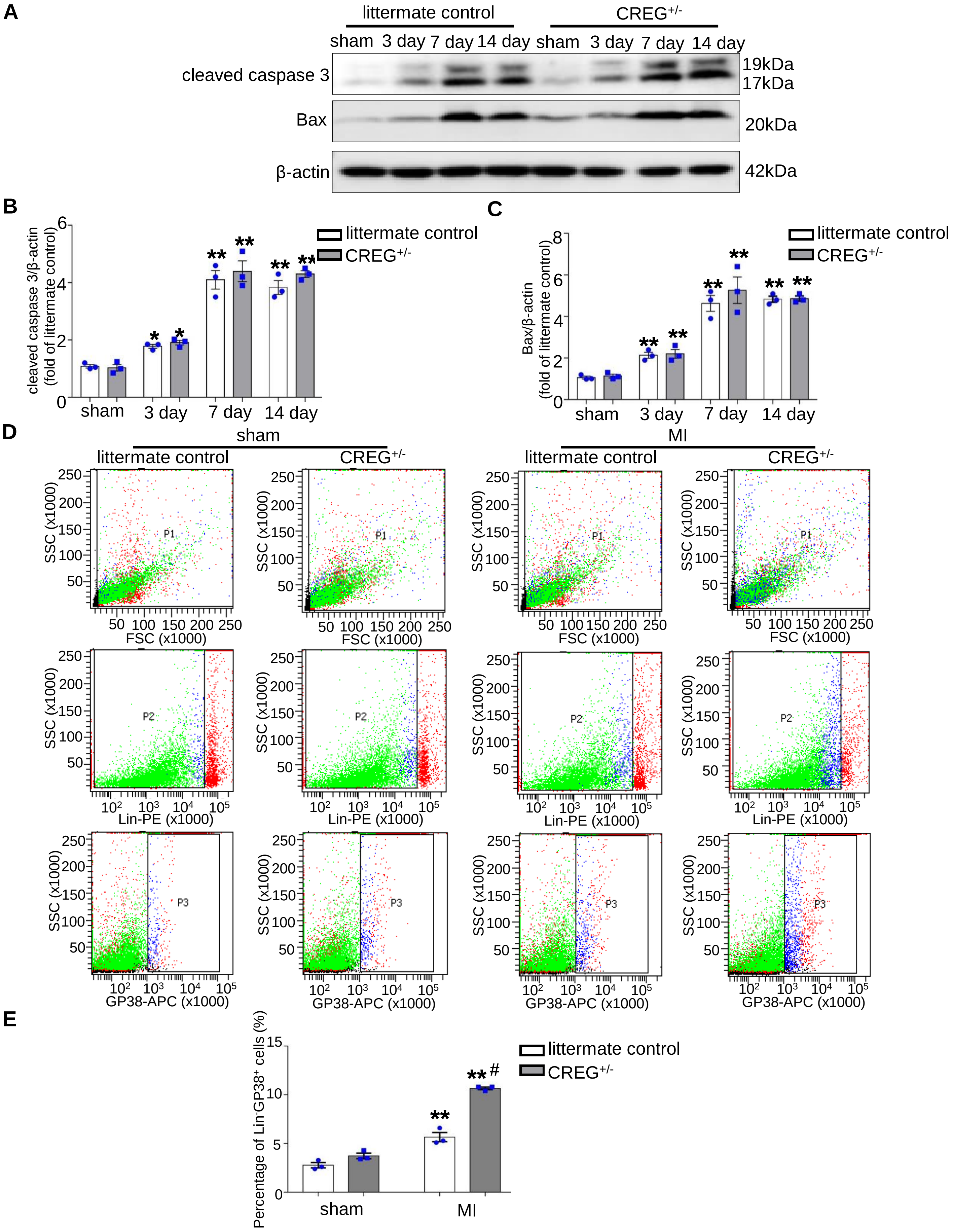

Figure S3. The cardiomyocytes apoptosis and cardiac fibroblasts proliferation in the border zone of CREG$^{+/-}$ mice and littermate control mice following MI

A-C. Western blotting of cleaved caspase 3 and Bax in the border zone of CREG$^{+/-}$ mice and littermate control mice on days 3, 7 and 14 following MI. **D, E.** Flow cytometry for the percentage of Lin$^-$GP38$^+$ fibroblasts in the myocardium of CREG$^{+/-}$ mice and littermate control mice on day 14 following MI. N=3 each group. **P<0.01 vs. sham group, $^{\#}$P<0.05 vs. littermate control-MI.

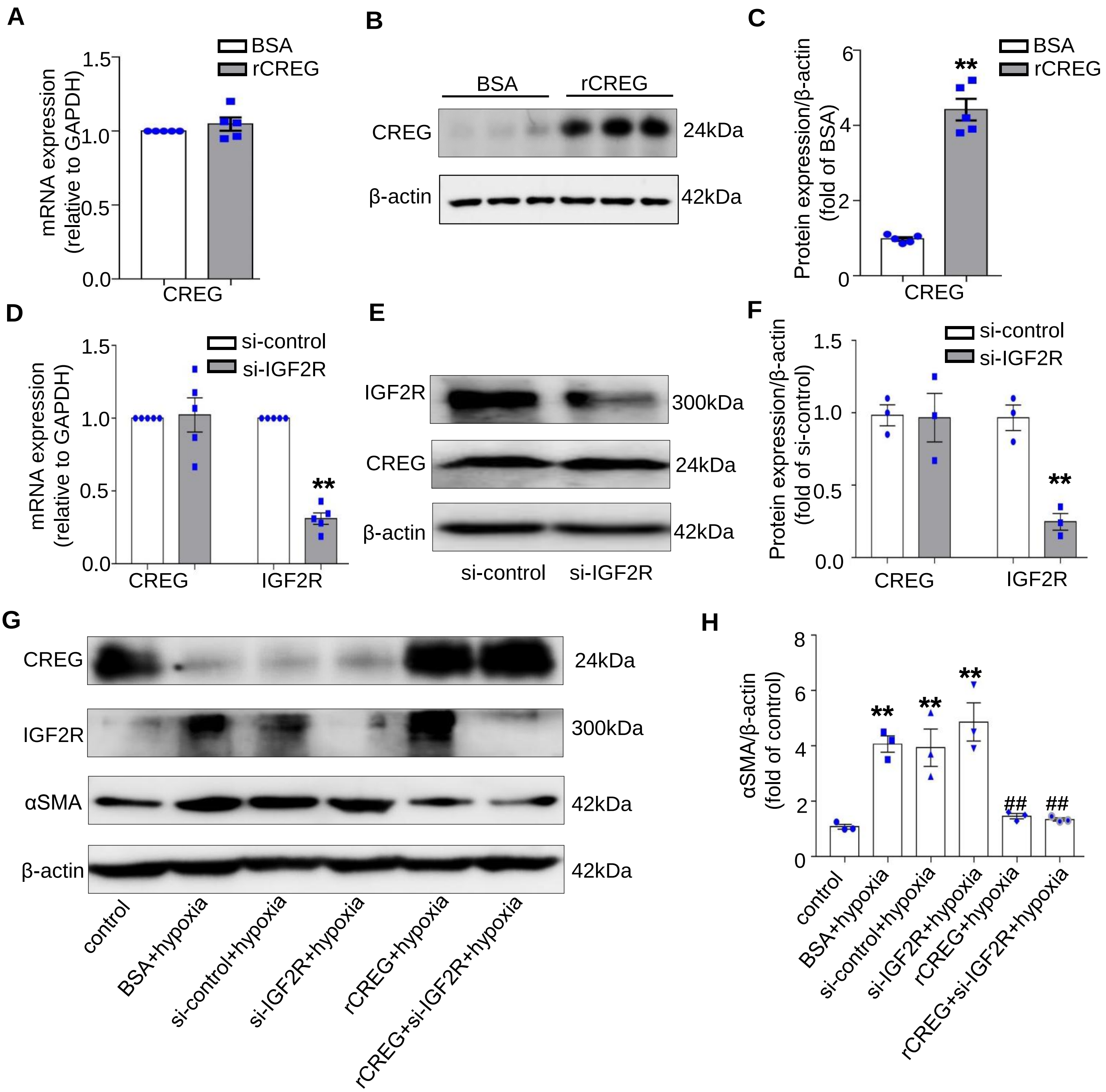

Figure S4. The effect of CREG on hypoxia-induced cardiac fibroblast activation was independent of IGF2R

A. *CREG* mRNA expression in CREG recombinant protein (rCREG) stimulated cardiac fibroblasts. **B, C**. CREG protein expression in rCREG stimulated cardiac fibroblasts. **D**. Real-time PCR of IGF2R knockdown on the mRNA expressions of *IGF2R* and *CREG*. **E, F**. Western blotting of IGF2R knockdown on the protein expressions of IGF2R and CREG. **G, H**. Western blotting of IGF2R knockdown on αSMA expression induced by hypoxia. N=3-5 each group. **P<0.01 vs. BSA or si-control or control group; $^{##}$P<0.01 vs. si-control+hypoxia group.

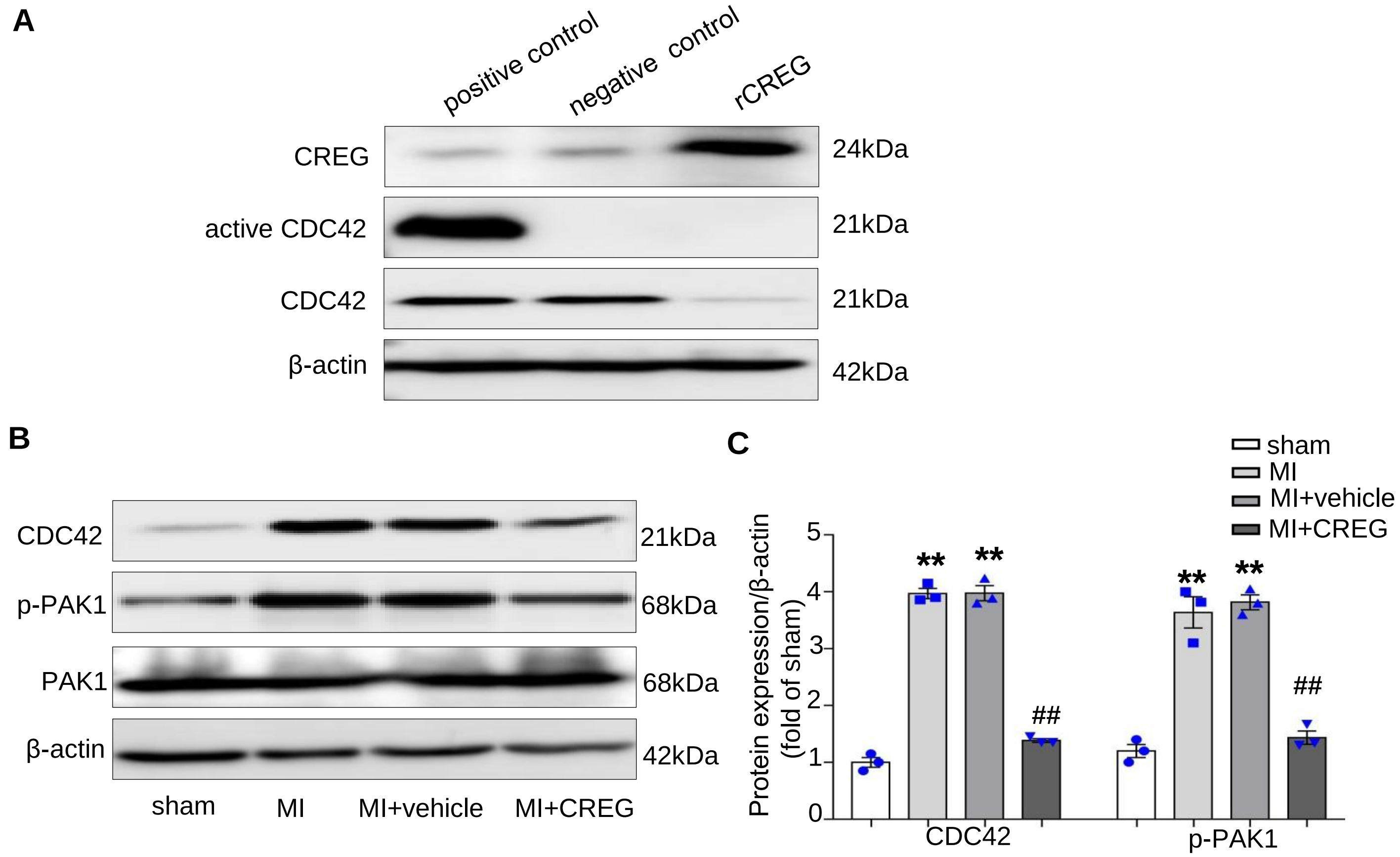

Figure S5. **Effect of CREG recombinant protein (rCREG) on the CDC42 activity and expression *in vitro* and *in vivo***

A. Pull-down assay to determine of the effect of rCREG on the CDC42 activity in mouse cardiac fibroblasts under normoxia. Mouse cardiac fibroblasts were stimulated with rCREG (5 μg/ml) for 24 h. **B, C**. Western blotting for CDC42 and pho-PAK1 in the border zone of myocardium on post-MI day 14. N=3 each group. **P<0.01 vs. sham group; ##P<0.01 vs. MI+vehicle group

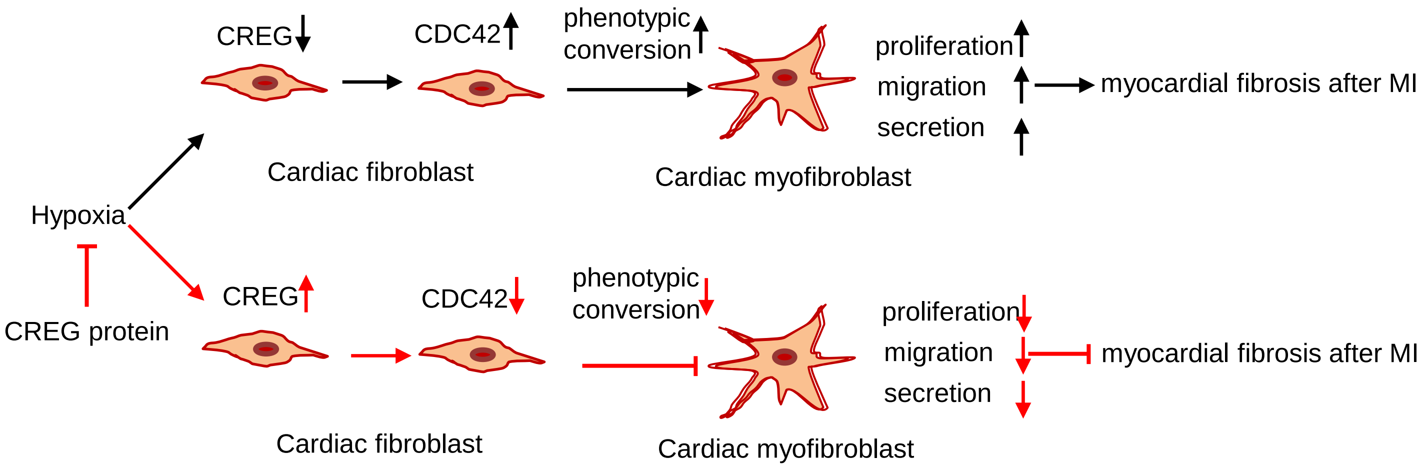

Figure S6. Schematic illustration of the proposed mechanism of CREG in cardiac fibroblast activation. Hypoxia could increase cardiac fibroblast activation by inhibiting CREG expression, which leading to myocardial fibrosis post myocardial infarction (MI). CREG recombinant protein could reverse the effect of hypoxia in the phenotypic conversion of cardiac fibroblasts during myocardial infarction, by inhibiting CDC42 expression.

Table S1. CREG deficiency aggravated the damage of left ventricular systolic function on post-MI day 14

	**littermate control**		**CREG$^{+/-}$**	
	sham	MI	sham	MI
**LVAWd (mm)**	0.97±0.05	0.62±0.08**	1.05±0.06	0.56±0.06**
**LVAWs (mm)**	1.43±0.07	0.69±0.10**	1.49±0.07	0.70±0.12**
**LVIDd (mm)**	4.03±0.06	5.10±0.10**	3.85±0.11	5.42±0.16$^{**\,\#}$
**LVIDs (mm)**	2.80±0.06	4.60±0.11**	2.67±0.08	5.12±0.15$^{**\,\#\#}$
**LVPWd (mm)**	0.78±0.08	0.77±0.05	0.80±0.04	0.52±0.06 $^{**\,\#\#}$
**LVPWs (mm)**	1.15±0.10	0.93±0.05	1.09±0.04	0.58±0.07$^{**\,\#\#}$

LV, left ventricular; MI, myocardial infarction; LVAWd, LV anterior wall during diastole; LVAWs, LV anterior wall during systole; LVIDd, LV internal diameter during diastole; LVIDs, LV internal diameter during systole; LVPWd, LV posterior wall during diastole; LVPWs, LV posterior wall during systole; EF, ejection fraction; FS, fractional shortening. **P<0.01 vs. sham, $^{\#}$P<0.05, $^{\#\#}$P<0.01 vs. littermate control-MI.

Table S2. CREG exogenous overexpression improved left ventricular systolic function on post-MI day 14

	sham	MI	MI+vehicle	MI+CREG
**LVAWd (mm)**	1.08±0.06	0.64±0.07**	0.64±0.08**	0.77±0.07**
**LVAWs (mm)**	1.49±0.75	0.70±0.09**	0.69±0.09**	0.92±0.10**
**LVIDd (mm)**	3.83±0.14	5.08±0.0.10**	5.00±0.12**	4.78±0.09**
**LVIDs (mm)**	2.69±0.12	4.59±0.11**	4.55±0.13**	4.11±0.10** ##
**LVPWd (mm)**	0.77±0.05	0.76±0.04	0.78±0.05	0.72±0.03
**LVPWs (mm)**	1.06±0.04	0.90±0.05	0.91±0.05	0.92±0.04

LV, left ventricular; MI, myocardial infarction; LVAWd, LV anterior wall during diastole; LVAWs, LV anterior wall during systole; LVIDd, LV internal diameter during diastole; LVIDs, LV internal diameter during systole; LVPWd, LV posterior wall during diastole; LVPWs, LV posterior wall during systole; EF, ejection fraction; FS, fractional shortening. **P<0.01 vs. sham, ##P<0.01 vs. MI+vehicle.
